# *Trametes meyenii* possesses elevated dye degradation abilities under normal nutritional conditions compared to other white rot fungi

**DOI:** 10.1186/s13568-014-0074-3

**Published:** 2014-09-30

**Authors:** Peter R Chenaux, Narisa Lalji, Daniel D Lefebvre

**Affiliations:** 1 Department of Biology, Queen’s University, Kingston K7L 3 N6, ON, Canada

**Keywords:** White-rot fungi, Dye decolouration, Trametes, Laccase, Manganese peroxidase, Manganic chelation

## Abstract

Several species of white-rot fungi were investigated for their utility in prolonged decolouration of the recalcitrant sulfonated azo dye, amaranth. *Trametes pubescens, T. multicolor, T. meyenii and T. versicolor* decoloured amaranth azo-dye best on low-nitrogen agar-solidified media whereas *Bjerkandera adusta* and *Phlebia radiata* were most effective in low nitrogen medium supplemented with manganese. *Trametes cotonea* did not decolour effectively under any condition. The decolouring *Trametes* species were also effective in liquid culture whereas *B. adusta* and *P. radiata* were not. *Trametes meyenii, T. pubescens* and *T. multicolor* were equal to or better than commonly employed *T. versicolor* at decolouring amaranth. This is the first study to show the dye decolouration potential of *T. meyenii*, *T. pubescens*, and *T. multicolor*. Supplementing with Mn(II) increased assayable manganese peroxidase activity, but not long-term decolouration, indicating that laccase is the main decolourizing enzyme in these *Trametes* species. This appears to be because of inadequate Mn^3+^ chelation required by manganese peroxidase because adding relatively low amounts of malonate enhanced decolouration rates. The ability of *Trametes meyenii* to simultaneously decolour dye over prolonged periods of time while growing in relatively nutrient-rich medium appears to be unique amongst white-rot fungi, indicating its potential in wastewater bioremediation.

## 1 Introduction

One of the largest sources of environmental pollutants is the textile industry, which can produce over 800 kilo tonnes of dye annually, with 90% of the waste discharged into the environment (Nigam et al. [2000]; Hessel et al. [2007]; Martin et al. [2012]). The strong structural integrity and toxic nature of dyes poses risk to flora, fauna and human populations (Chagas and Durrant [2001]). The four main chromophoric groups include azo, anthraquinone, triarylmethane and phthalocyanine, with azo dyes accounting for over 50% used in the textile industry (Heinfling et al. [1998a]; Reddy and Mathew [2001]). Azo dyes are generally also the most difficult to degrade (Toh et al. [2003]; Dafale et al. [2010]). Amaranth, a sulfonated compound, was employed as a recalcitrant azo dye in this study.

White-rot fungi are able to degrade lignin, a complex plant biopolymer (Evans and Hedger [2001]). They employ relatively non-specific enzymes that are also able to attack a wide range of pollutants including textile dyes (Archibald et al. [1997]; Van Aken et al. [1999]; Pointing [2001]; Ramsay and Goode [2004]). Even though *Trametes versicolor* (Swamy and Ramsay [1999b]) and some other white-rot fungi (Heinfling et al. [1998b]; Levin et al. [2002]) do produce these enzymes under nutrient-rich conditions, it is generally accepted that effective degradation occurs during the induction of secondary metabolism only when carbon or nitrogen supplies are low (Archibald et al. [1997]; Kaal et al. [1995]; Swamy and Ramsay [1999a]; Hatvani and Mécs [2002]). It would, however, be more desirable from a bioremediation perspective to utilize well nourished growing organisms to decolor dyes for prolonged periods of time.

Manganese peroxidase (MnP) is the most common ligninolytic peroxidase as it is produced by almost all white-rot basidiomycetes (Morgenstern et al. [2008]; Tomsovsky et al. [2009]), and laccase occurs in almost all wood- and litter-transforming basidiomycetes (Wesenberg et al. [2003]). These enzymes play major roles in decolorization processes in the fungal genus *Trametes* where they can be expressed to some degree under primary as well as secondary metabolism (Libra et al. [2003]). They are also the main lignin modifying enzymes produced by *T. versicolor* during decoloration of amaranth dye (Swamy and Ramsay [1999b]; Champagne and Ramsay [2005]). Although laccase activity predominates under well nourished conditions, it may not be able to decolorize dye in the absence of MnP (Wesenberg et al. [2003]; Viswanath et al. [2014]). Because of their common occurence and enzyme efficiencies (Morgenstern et al. [2008]; Wesenberg et al. [2003]; Tomsovsky et al. [2009]) and the fact that normal culture conditions for fungi do not induce lignin peroxidase activity (Swamy and Ramsay [1999b]), enzyme investigations were limited to MnP and laccase in this study.

Although there have been several studies on dye degradation by *T. versicolor*, many of the estimated fifty *Trametes* species (Kirk et al. [2009]) have not been investigated. This provides a large resource within which to search for species with desireable bioremediation properties. In the current study we investigate white-rot fungal species, including five species of *Trametes*, to determine how well they degrade amaranth and produce MnP and laccase in response to different nutritional treatments. Notable species of the genus *Trametes* were distinguished by their ability to effectively degrade dye over extended periods of time, and to do so, *Trametes meyenii* in particular, did not require nutrient deprivation.

## 2 Materials and methods

### 2.1 Culture maintenance and media

Seven species of fungi (Table 1) were maintained as stocks in 100 x 15 mm petri plates containing 15 mL of modified Kirk’s medium (Kirk and Fenn [1982]) with 3% (w:v) malt agar at 4°C and pH 5.0. The modified Kirk’s medium consisted of 10 g L^-1^ glucose, 1.2 g L^-1^ ammonium tartrate, 0.05 g L^-1^ MgSO_4_.7H_2_O, 0.01 g L^-1^ CaCl_2_.2H_2_O, 0.20 g L^-1^ K_2_HPO_4_, 1 μg L^-1^ thiamine, 1 mL L^-1^ trace mineral solution and 15 g L^-1^ agar. The trace mineral solution contained 1 g L^-1^ NaCl, 0.5 g L^-1^ MnSO_4_.H_2_0, 0.1 g L^-1^ CoSO_4_, 0.1 g L^-1^ FeSO_4_.7H_2_O, 0.1 g L^-1^ ZnSO_4_.7H_2_O, 82 mg L^-1^ CaCl_2_, 10 mg L^-1^ CuSO_4_.5H_2_O, 10 mg L^-1^ NaMoO_4_.2H_2_O, 10 mg L^-1^ H_3_BO_3_, and 0.1 g L^-1^ ethylene diamine tetraacetic acid at pH 5.0.

**Table 1:** White-rot fungal strains

**Species**	**Strain code**
*Bjerkandera adusta*	ATCC^a^ MYA-264
*Phlebia radiata*	ATCC 64658
*Trametes cotonea*	CBS^b^ 352.80
*Trametes meyenii*	CBS 453.76
*Trametes multicolor*	VIAM^c^ MB 49
*Trametes pubescens*	CBS 396.90
*Trametes versicolor*	ATCC 20869

^a^ATCC, American Type Culture Collection, Manassas, USA; ^b^CBS, Centraalbureau voor Schimmelcultures, Utrecht, The Netherlands; ^c^VIAM, Culture Collection of the Institute of Applied Microbiology, University of Agricultural Sciences, Vienna, Austria.

### 2.2 Influence of culture parameters on growth and amaranth decoloration on agar-solidified media

Circular plugs measuring 0.5 cm diam. were taken from stock plates using the wide end of a sterilized 200 μL pipet tip (Advantech AD200Y-K, Diamed Lab Supplies Inc., Mississauga, Canada) and placed in the center of fresh plates and grown at 28°C. After 4 days fresh plugs were placed on experimental media shown in Table 2. Amaranth was added at 50 ppm (83 μM) unless otherwise stated. Plates were kept at 28°C and fungal growth and dye decoloration, where appropriate, were measured daily until either fungal growth or decoloration reached the edge of the plate. Growth and decolored zones were determined by measuring their areas on the plates. Experiments were performed in octuplicate.

**Table 2:** Modifications made to standard Kirk’s medium

**Medium**	**Details**
*Low-N Kirk’s* (1.2 mM)	Basic Kirk's containing 0.22 g L^-1^ ammonium tartrate.
*High-N Kirk’s* (12 mM)	Basic Kirk's containing 2.2 g L^-1^ ammonium tartrate.
*Low-Glu Kirk's*	Basic Kirk's containing 1 g L^-1^ glucose.
*Low-N/Low-Glu Kirk's*	Basic Kirk's containing 1 g L^-1^ glucose & 0.22 g L^-1^ ammonium tartrate.
*Low-N Kirk's* + 200 μM Mn(II)	Basic Kirk's containing 0.22 g L^-1^ ammonium tartrate & 0.034 g L^-1^ MnSO_4_.H_2_O.

### 2.3 Influence of culture parameters on amaranth decoloration and enzyme activity in liquid media

Ten 0.5 cm diameter circular agar plugs from 4 day old petri plate cultures were added to each 500 mL Erlenmeyer flask containing 100 mL of Kirk’s medium. These were grown at 28°C with rotary shaking at 100 rpm. The Kirk’s medium used followed the same recipe as for the agar-solidified medium without the agar and supplementation with 20 mM 2,2-dimethylsuccinate to act as a buffer (Swamy and Ramsay [1999a]). Amaranth treatment was performed as follows. After 5 days the medium was decanted and the fungal pellets were resuspended in 100 mL of fresh Kirk’s medium containing 0.12 mM ammonia tartrate (low-N medium) and 83 μM (50 ppm) amaranth. After 4 days, this medium was replaced with that of the same composition and samples were taken at increasing time intervals to determine decoloration of amaranth and enzyme activities. Where appropriate, the effect of Mn was by the addition of 200 μM MnSO_4_.H_2_O. All experiments were performed in duplicate with four replicates for each treatment.

Long term decoloration experiments were initiated by adding 5 mL wet volume of fungal biomass from 5 day old culture in liquid Kirk’s medium without amaranth to 100 mL culture flasks containing various media. Experiments were started at 83 μM amaranth which was replenished after complete decolorations occurred as often as required.

### 2.4 Assays of decoloration and enzyme activities

One mL samples were taken at each time point and immediately replaced with Kirk’s medium containing no glucose or ammonia tartrate. Amaranth dye concentrations were determined spectrophotometrically at 523 nm. An O.D._523_ of 1.0 per cm corresponded to an amaranth concentration of 41.5 μM. MnP activity was measured by monitoring specific oxidation of Mn^2+^ to chelated Mn^3+^ (Wariishi et al. [1992]). One unit (U) of activity equals 1 μmol Mn^2+^ oxidized per minute at 25°C and pH 4.5. Laccase activity assays were performed by measuring the oxidation of 2,2’-azino-bis(3-ethylbenzthiazoline-6-sulfonic) acid (ABTS) at 420 nm (ε_420_ = 36,000 M^-1^ cm^-1^) (Johannes and Majcherczyk [2000]). One unit (U) of activity equals 1 μmol ABTS oxidized per minute at 25°C and pH 5.0. These assays contained 50 mM sodium acetate buffer and 0.2 mM ABTS. All assays were performed in quadruplicate using 96-well microtiter plates and measured with a Spectra Max Plus Spectrophotometer (Molecular Devices, Sunnyvale, CA). One direction ANOVA with a Tukey-Kramer HSD post hoc test was performed using JMP 10.0 software (SAS Incorporated, Toronto, Canada).

## 3 Results

### 3.1 Effect of nutrition on decoloration on agar-solidified media

The effect of medium composition on fungal abilities to decolor the azo-dye, amaranth in seven different species of white-rot fungi (Table 1) was tested on different variations of nutrient media (Table 2).

Decoloration abilities after 6 days growth on agar plates are presented in Table 3. After a lag of 3-4 days all species except for *Trametes cotonea* decolored amaranth. Complete dye decoloration occurred for *Phlebia radiata* on low N Kirk’s with Mn, and for *Trametes meyenii, Trametes pubescens and Trametes multicolor* on low N/low glucose Kirk’s. Significantly, *T. meyenii* also decolored completely on full nutrient Kirk’s medium.

**Table 3:** Decoloration of amaranth by white-rot fungi grown on agar-solidified media

**Species**	**Kirk’s**	**LG Kirk’s**	**HN Kirk’s**	**LN Kirk’s**	**LN/LG Kirk’s**	**LN Kirk’s + Mn**
*B.adusta*	+	++	-	++	++	+++
*P.radiata*	+	+	+	++	-	++++
*T.cotonea*	-	-	-	-	-	-
*T.meyenii*	++++	+++	+++	+++	++++	++
*T.pubescens*	++	+++	+++	+++	++++	+++
*T.multicolor*	+	-	+	+++	++++	++
*T.versicolor*	+	-	+	+++	+++	+++

L, low; H, high; G, glucose; N, nitrogen; -, no decolouration; +, low decolouration; ++, intermediate decolouration; +++, strong decolouration; ++++, complete decolouration. Results were consistent over four independent experiments.

### 3.2 Decoloration studies in liquid cultures

**Figure 1:**
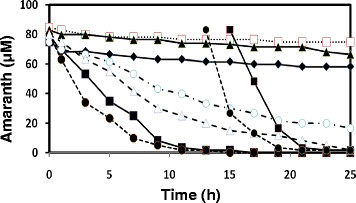
**Amaranth decolouration by 9-day-old cultures in low-N Kirk’s liquid medium.***B. adusta***(♦**), *P. radiata* (**□**), *T. cotonea* (**▲**), *T. meyenii* (**○**), *T. pubescens* (**●**), *T. multicolor* (**■**), *T. versicolor* (∆)*.* S.D. always less than 4% (n = 5).

**Figure 2:**
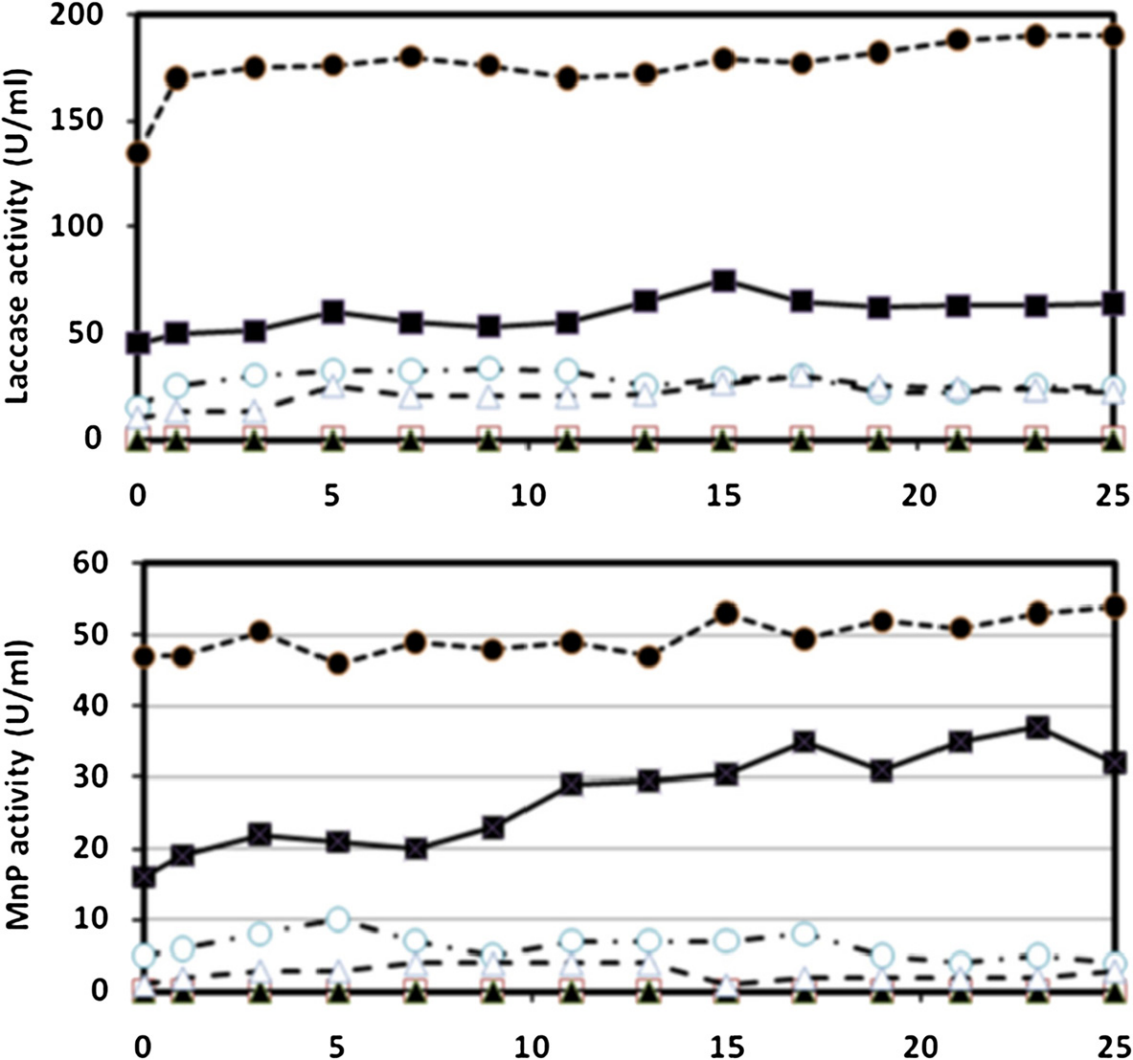
**Laccase and MnP activities by 9-day-old cultures in low-N Kirk’s liquid medium.***B. adusta***(♦**), *P. radiata* (**□**), *T. cotonea* (**▲**), *T. meyenii* (**○**), *T. pubescens* (**●**), *T. multicolor* (**■**), *T. versicolor* (∆)*.* S.D. always less than 5% (n = 5). *B. adusta*, *P. radiata* and *T. cotonea* all displayed negligible enzyme activities.

Seven species of white-rot fungi were grown in liquid medium containing amaranth for four days. Low-N Kirk’s was chosen because most species (6 of 7) had substantial decoloration capabilities on agar-solidified medium of this composition (Table 3). All species began decoloration immediately (Figure 1). *Phlebia radiata*, *Bjerkandera adusta*, and *T. cotonea* decolored amaranth only slightly which may be attributed to sorption to biomass rather than by enzymatic degradation. This is corroborated by the lack of detectable MnP and laccase activities in these species (Figure 2). The remaining species did not absorb dye. *Trametes multicolor* and *T. pubescens* decolored the best and both were able to decolor a second addition of dye within a 24 hour period, with the rates of the 2nd decoloration being approximately double that of the first exposures. *Trametes versicolor* and *T. meyenii* were able to achieve approx. 94 and 77 percent decoloration within 24 hours, respectively.

Secreted laccase and MnP were detected for the four species that showed the highest decoloring abilities, with laccase as the main lignin modifying enzyme (Figure 2). *Trametes pubescens* had the most laccase activity, which was three times that of the next best laccase producing species, *T. multicolor. Trametes pubescens* also had the highest MnP production, which was twice that of *T. multicolor*. Enzyme activities for lignin peroxidase, versatile peroxidase and dye-decolorizing peroxidase were not detected under the culturing conditions employed.

### 3.3 Sequential decoloration experiments

The four *Trametes* species that efficiently decolored amaranth dye in liquid medium were investigated to determine their respective most appropriate nutrient conditions for decoloration. A summary of these decoloration and enzyme activity characteristics are given in Table 4.

**Table 4:** **Decolouration and enzyme characteristics of** ***Trametes*** **species cultured in liquid media for 29 days**

	**Decoloration**				**Enzyme activities**					
	**Initial decoloration period (days)**	**Number of decolorations**	**Rate of decoloration at 29 days**	**Average time for decoloration (days)**	**Mn Peroxidase**			**Laccase**		
					**Maximum (U/ml)**	**Day at maximum**	**Average (U/ml)**	**Maximum (U/ml)**	**Day at maximum**	**Average (U/ml)**
Trametes versicolor										
Kirks	4	8	Low	2.75	18.2	15	8.0	90.5	8	55.0
LN Kirks	3	19	Inter	1.50	8.3	6	5.3	91.8	6	34.1
LN Mn Kirks	3	16	Low	1.38	25	4	11.0	72.2	5	40.4
Trametes multicolor										
Kirks	3	21	Inter	1.29	22.5	4	11.1	84.1	6	42.7
LN Kirks	3	24	High	1.25	38.9	5	6.5	87.4	5	40.2
LN Mn Kirks	3	20	Low	1.10	50.0	5	18.3	84.6	6	35.0
Trametes pubescens										
Kirks	3	19	Inter	1.39	10.4	17	4.5	78.6	21	26.1
LN Kirks	3	26	High	1.08	11.3	21	5.0	80.3	22	29.8
LN Mn Kirks	3	20	Low	1.30	19.1	14	5.2	77.2	17	26.3
Trametes meyenii										
Kirks	4	23	High	1.26	20.5	15	7.3	88.1	17	31.5
LN Kirks	4	10	inter	2.91	20.8	6	4.8	86.7	8	39.6
LN Mn Kirks	6	8	Inter	3.49	46.3	11	15.1	90.3	29	36.1

Normal (Kirk’s), low nitrogen (LN Kirk’s), and low nitrogen supplemented with Mn (LN Mn Kirk’s); for more details see Table 2. Each value is an average of five separate experiments. S.D. always less than 4% (n = 5).

The greatest number of repeat decolorations occurred with *T. pubescens* grown in low-N Kirk’s, which was able to decolor 26 sequential daily additions of 83 μM amaranth over a 29 day period. This species also had the highest level of laccase and MnP activities in this medium. The addition of Mn(II) or sufficient N resulted in 20 and 19 sequential decolorations, respectively, with reduced decoloration rates by day 20 in both. This decrease in decoloration rate was not reflected in measured enzyme activities which remained elevated (data not shown).

*Trametes multicolor* grown in low-N Kirk’s was able to decolor 24 sequential additions of amaranth over 29 days. As with *T. pubescens*, this also corresponded to the highest levels of MnP and laccase activity in that species. The addition of Mn(II) decreased these enzymes’ activities, and this was even more pronounced in normal Kirk’s medium. After day 5 both laccase and MnP activities declined (data not presented). Despite this, decoloration rate was maintained until day 23 with most daily pigment additions being completely removed between 5 and 24 hours. This trend was most notable in low-N Kirk’s, which was still able to decolor somewhat at day 29 despite there being very little measurable laccase or MnP activity.

*Trametes versicolor* also had its highest number of decolorations under low-N conditions, decoloring 20 sequential additions in 29 days. This also did not correspond to the highest activity of laccase and MnP. The highest enzyme activity was found in normal Kirk’s medium, where only 9 sequential decolorations occurred. The addition of 200 μM Mn(II) to the medium doubled MnP but decreased laccase activity.

*Trametes meyenii* was the only species that had higher decoloration rates in the higher-N containing Kirk’s medium. It decolored over twice as many sequential additions of amaranth in contrast to the low-N treatment. Both laccase and MnP activities were also highest for this condition. Even though the addition of Mn(II) to the low-N culture medium doubled MnP activity in *T. meyenii*, there was no concomitant increase in decoloration rate. In fact, the initial lag phase took the longest in this condition, with 7 days to complete the first decoloration as opposed to 5 days for the Kirk’s treatments.

### 3.4 Decoloration in the presence of malonate

**Figure 3:**
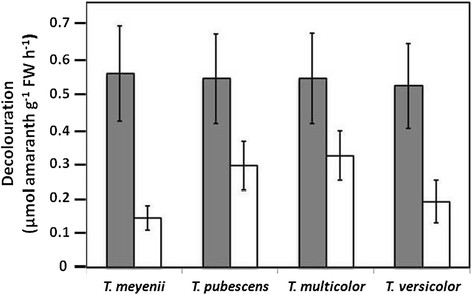
**Rate of amaranth decolouration in the presence and absence of malonate by four*Trametes*species grown in low-N Kirk’s.** Presence and absence of 50 μM sodium malonate. All values are means ± S.D. (n = 5).

Rates of decoloration increased significantly for all species of *Trametes* when 50 μM sodium malonate was added to cultures that had been pretreated in low-N Kirk’s (Figure 3). However, a comparison between species found no significant differences among the resultant rates of decoloration on a per fresh wt basis.

## 4 Discussion

The bioremediation of chemical contaminants requires the identification of appropriate species that are easily maintained in active condition. It is commonly accepted that stress conditions such as nutrient deprivation with their associated growth inhibition must elicit secondary metabolism before appropriate enzymes are activated in white-rot fungi (Archibald et al. [1997]; Hatvani and Mécs [2002]; Swamy and Ramsay [1999b]; Kaal et al. [1995]; Tatarko and Bumpus [1998]; Tenuta and Lazarovits [2002]). However, the provision of highly effective degradative processes by healthy and actively growing organisms would be more practical and efficaceous. This could also help to overcome the limited utility of fungal cultures for dye decolorization (Kokol et al. [2007]).

Screening for effectiveness of dye decoloration on agar-solidified media indicated that *T. cotonea* had little activity and that of both *B. adusta* and *P. radiata* were much less effective than the four other tested *Trametes* species (Table 3). This might be attributed to the strategies of the individual species with respect to natural substrate degradation (Barrasa et al. [2014]). Furthermore, *P. radiata*, *B. adusta*, and *T. cotonea* decolored amaranth only slightly in liquid media through a process that involved sorption to their biomass (Figure 1). This was characterized by darkening of their mycelia with little or no secreted enzyme production (Figure 2). Results on agar-solidified media indicated that *P. radiata* and *B. adusta* are able to decolor amaranth and studies by other researchers using liquid culture have shown decoloration (Kaal et al. [1995]; Robinson et al. [2001]; Arora and Gill [2001]). However, submerged cultures can display reduced decoloration because of low enzyme levels, and peroxidase activities are generally only optimal at high O_2_ tension. Laccase, on the other hand, can be enhanced at relatively low O_2_ (Wesenberg et al. [2003]).

*Trametes meyenii, T. pubescens* and *T. multicolor* were equal to or better at decoloring amaranth than commonly employed *T. versicolor*. Of these, *T. pubescens* has been used to a limited extent in bioremediation of phenols (Ryan et al. [2005]) and wine wastewater (Strong and Burgess [2007]), and *T. multicolor* has been studied in the context of PCB degradation (Köller et al. [2000]). Direct comparisons in decoloration and enzyme activites between these *Trametes* species has not been previously made.

*Trametes meyenii* had the highest rate of growth at 8% over the next best grower, *T. multicolor*, and was the only species to decolor best on normal Kirk’s medium where it possessed prolonged activity. This supports an important role for laccase in the decoloration of amaranth by *T. meyenii* (Table 4). Laccase is produced during growth in *T. versicolor*, while peroxidases are produced during secondary metabolism (Wong and Yu [1999]). Laccase production is also stimulated by low glucose (Tavares et al. [2005]) and the presence of nitrogenous dye in this species (Casas et al. [2013]), and dye degradation has been attributed solely to laccase in the white-rot fungus *Datronis* sp. (Vaithanomsat et al. [2010]). The present study is the first to test dye decoloration and associated enzyme production in *T. meyenii*.

Liquid culturing more accurately reflects real waste-water treatment conditions. Low-N medium was tested initially because previous studies (Swamy and Ramsay [1999a]; Swamy and Ramsay [1999b]; de Jong et al. [1994]) and our investigations with agar-solidified media showed that this enabled all species to decolor dye, with the exception of *T.* cotonea. Unlike the situations for the remaining *Trametes* species, *P. radiata* and *B. adusta* were severely affected by oxygen limiting conditions of liquid media. *Trametes pubescens* and *T. multicolor* were the most efficient and displayed the highest levels of secreted laccase and MnP activity in low-N media. However, there was not always a correlation between measured enzyme activities and a species’ ability to decolor amaranth. High nitrogen has been previously shown to increase MnP (Kaal et al. [1995]) and laccase production without increasing decoloration (Robinson et al. [2001]). However, these other studies used very high ammonia that could cause stress in fungi (Tenuta and Lazarovits [2002]), thereby invoking secondary metabolism.

The most efficient process from a bioremediation perspective would be if white-rot fungi were able to decolor consecutive additions of dye over an extended period of time. Therefore the four efficient *Trametes* species in this study were investigated further to determine their ability to decolor successive additions of the dye. The activities of laccase and MnP enzymes were monitored to determine their contributions to the degradative process because of their common occurence in white-rot fungi (Morgenstern et al. [2008]; Wesenberg et al. [2003]; Tomsovsky et al. [2009]) and active roles in dye degradation under normal culture conditions, which does not appear to be the case for lignin peroxidase (Swamy and Ramsay [1999b]).

Laccase and MnP were the only detected decolorizing enzymes in the culture media. With respect to lignin peroxidase, it is known that the culturing conditions used in the present study for *Trametes* fungi do not result in lignin peroxidase activity (Swamy and Ramsay [1999b]). Furthermore, although DNA sequences for putative versatile and dye-decolorizing peroxidases exist in at least one of the fungal species of the presented study (Hofrichter et al. [2010]; Ruiz-Dueñas et al. [2007]), respective enzyme activities have yet to be demonstrated in this genus. For example, manganese-independent peroxidase (versatile peroxidase) was not detected in *T. versicolor* by Gavril et al. ([2007]). In addition, assaying for this enzyme (de Jong et al. [1992]) and dye-decolorizing peroxidase (Sugano et al. [2006]) did not detected any activity in the present study. The roles of laccase and MnP in the decoloration process is further supported by lag periods prior to decoloration corresponding to enzyme production in the culture media (Table 4). Once induced, degradation rates improved dramatically, with complete decoloration occurring in as little as 15 hours (Figure 1). However, dye decoloration by *T. versicolor* has been shown to be initially low regardless of the levels of measurable MnP and laccase (Swamy and Ramsay [1999b]). This is supported by our findings that enzyme activities did not closely correspond to rate of decoloration (Table 4), thus indicating that other important factors are required for prolonged decoloration.

Despite *T. versicolor* having the highest measurable maximum and average laccase activities in long-term experiments, it was out performed by *T. multicolor* and *T. pubescens* in decoloration in low nitrogen. This could be in part because MnP activity was higher in *T. multicolor*, but that of *T. pubescens* was lower despite other research to the contrary (Strong and Burgess [2007]). *Trametes meyenii* was exceptional because it possessed the longest period of rapid decoloration under higher nitrogen nutrition even though its enzyme activities were similar to those of *T. versicolor* and T. *multicolor*.

Supplying white-rot fungi with Mn(II) in low N Kirk’s led to increases in measurable MnP production, but no improvement in long-term decoloration in all four *Trametes* species*.* This may indicate that either MnP is not the main decolorizing enzyme, or other necessary factors are more readily produced at low nitrogen.

It has been shown that despite high levels of extracellular enzymes, decoloration diminishs as glucose is depleted and is restored by its replenishment (Swamy and Ramsay [1999a]). This could act as a general fixed carbon source required for the production of factors needed by the enzymes or more specifically, it could be a direct precursor of H_2_O_2_ via glucose-2-oxidase (Champagne and Ramsay [2005]; Sen et al. [2012]). We found that a relatively low concentration of malonate that would not be a significant source of carbon nutrition enhanced decoloration rates to the same magnitude on a per g FW basis in all four *Trametes* species when they were grown in low-N Kirk’s medium (Figure 3). Manganese peroxidase oxidizes Mn^2+^ to Mn^3+^ which because of its instability, must chelate with an organic compound such as malonate (Schlosser and Hofer [2002]; Kersten and Cullen [2007]; Lundell et al. [2010]; Liu et al. [2011]). These chelators can act as low-molecular mass mediators (Higuchi [2004]) that react with each other to eventually form peroxyl radicals (Gianfreda et al. [1999]) able to attack a wide range of compounds non-specifically (Wariishi et al. [1988]; Kuan and Tien [1993]; Watanabe et al. [2000]).

White-rot fungal species respond to nutrient conditions indicative of their specific ecological niches (Lundell et al. [2010]; Tuor et al. [1995]; Hiscox et al. [2010]). This is the first study to show the dye decoloration potential of *T. meyenii*, *T. pubescens*, and *T. multicolor*. All three of these species possessed a strong capacity for prolonged dye decoloration by comparison with the more commonly studied *T. versicolor*. Enzyme activity for both laccase and MnP occurred over the decolorizing periods in all of these species. However, it appears that manganic chelation limited the degradative process and that the most efficient conditions for any given species may be that which enables the organisms to adequately produce chelating compounds. Studies with *T. meyenii* may help provide insight into the chelation phenomena because unlike other species it functions best when provided with full nutrient medium, a condition conducive to high metabolite production. In addition, more needs to be understood about the relative importance of secreted laccase and MnP enzymes. In some species of fungi, laccase and MnP have been shown to have an indirect interactive effect that enhances lignin depolymerization many times over that of individual enzymes, because of the production of H_2_O_2_ by laccase as a result of the oxidation of Mn^2+^ (Galliano et al. [1991]; Leonowicz et al. [1999]; [2001]). However, the contribution of laccase and MnP to amaranth decoloration appeared to be only additive in *T. versicolor* with MnP providing considerably more to the decoloration process (Champagne and Ramsay [2005]). It remains to be seen if this is the case for the other species of *Trametes* utilized in this study.

Of all the species tested, *T. meyenii* showed the most promise for industrial application purposes even though *T. multicolor* and *T. pubescens* functioned well in low-N medium. This is because *T. meyenii* possesses an elevated ability to decolor in higher-N environments, and most textile wastewaters contain relatively high levels of nitrogen, in part because of the nitrogenous structure of many dyes. This capacity to continue to simultaneously decolor dye over prolonged periods while growing in relatively nutrient-rich medium appears to be unique amongst white-rot fungi. The ecological significance of an ability to maintain lignolytic activity during higher nutritional states is also noteworthy.

## Competing interests

The authors declare that they have no competing interests.

## Authors’ contributions

PRC designed and performed decoloration and enzyme assays and helped draft the manuscript. NL designed and undertook decoloration studies in the presence of chelators. DDL conceived and designed the study, supervised the research group, provided funding support, drafted and revised the manuscript. All authors approved the final manuscript.
